# 7-O-Galloyltricetifavan: a promising natural radical scavenger

**DOI:** 10.1098/rsos.211906

**Published:** 2022-06-22

**Authors:** Le Trung Hieu, Tran Thi Van Thi, Nguyen Thi Hoa, Adam Mechler, Quan V. Vo

**Affiliations:** ^1^ University of Sciences, Hue University, Thua Thien Hue 530000, Vietnam; ^2^ The University of Danang – University of Technology and Education, Danang 550000, Vietnam; ^3^ Department of Chemistry and Physics, La Trobe University, Victoria 3086, Australia

**Keywords:** flavonoid, density functional theory, antiradical activity, antioxidant, kinetics

## Abstract

7-O-Galloyltricetifavan (**7OGT**), a natural flavonoid, is isolated from the leaves of *Pithecellobium clypearia*. The compound exhibits a variety of biological activities. This study details the evaluation of the HOO^•^ antiradical activity of **7OGT** by quantum chemistry calculations. The HOO^•^ trapping activity of **7OGT** in the gas phase (reference state) was discovered to follow the formal hydrogen transfer mechanism with a rate constant of *k* = 4.58 × 10^8^ M^−1^ s^−1^. In physiological environments, **7OGT** is predicted to be an excellent HOO^•^ radical scavenger with *k*_overall_ = 2.65 × 10^8^ and 1.40 × 10^4^ M^−1^ s^−1^ in water and pentyl ethanoate solvents, respectively. The HOO^•^ antiradical activity of **7OGT** in water at physiological pH is approximately 2000 times that of Trolox and substantially higher than that of other well-known natural antioxidants such as trans-resveratrol or ascorbic acid. Thus, **7OGT** is an excellent natural antioxidant in polar environments.

## 1. Introduction

**Figure 1.**
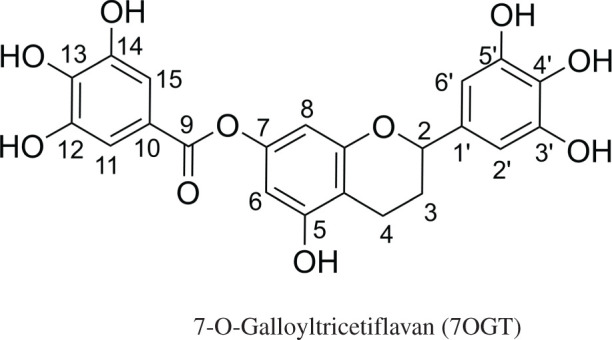
The structure of 7OGT.

7-O-Galloyltricetifavan (**7OGT**; figure 1), a natural flavonoid, was first isolated from the leaves of *Pithecellobium clypearia* [1–3]. **7OGT** is a flavan derivative that has antiviral properties against respiratory syncytial virus, influenza H1N1 virus, herpes simplex virus type 1 and coxsackie B3 virus as well as anti-inflammatory, anti-Alzheimer, anti-allergic and antioxidant properties [1–7]. Studies showed that **7OGT** has potent xanthine oxidase inhibition with an IC_50_ = 25.5 µmol l^−1^ [3] and inhibits soluble epoxide hydrolase enzymatic activity with IC_50_ values 10.0 ± 0.4 µM [5]. Thus, **7OGT** is indicated as a good natural antioxidant with the known neuroprotective activity that is believed to underpin the prevention of Alzheimer's disease [7].

Oxidative stress is now thought to play a role in several chronic diseases [8–10]. The ability of natural products to scavenge free radicals is an essential aspect of their anti-inflammatory, antibacterial and cancer-preventive properties, and it is the driving force behind the investigation of the antioxidant properties [11–13]. There is currently no information about the kinetics and mechanism of the HOO^•^ + **7OGT** in physiological conditions; however, computer calculations offer a convenient way to predict the antioxidant activity of organic compounds in physiological media [11,14,15]. Since **7OGT** has exhibited a broad range of potent biological activities, this study aims to delve into the underpinning antiradical activity of **7OGT** by a quantum chemical approach, using HOO^•^ as a model radical.

## 2. Computational methods

The density functional theory-based quantum chemical calculations used here had been outlined in a range of former works for modelling antioxidant activities of various compounds [16–20]. In brief, the M06–2X/6-311 ++ G(d,p)//M06-2X/6-31 + G(d) method was used to calculate thermodynamic parameters in the gas phase [21]. The kinetic calculations were performed at the M06-2X/6-311 ++G(d,p) level of theory, following the quantum mechanics-based test for overall free radical scavenging activity (QM-ORSA) protocol [22–24] with the SMD solvation model [25] for water and pentyl ethanoate solvents [11,14,17,19,26–35]. This protocol delivers results in reasonably good agreement with experimental data (*k*_calc_/*k*_exp_ ratio = 1–2.9) [11,26,36], and therefore, it is commonly used to assess the radical scavenging activity of natural and synthetic compounds [14,15,20,37,38].

Using the transition state (TS) theory at 298.15 K, 1 M standard state, the rate constant (*k*) was computed as follows [31–34,39]:

2.1 $$k=\sigma \kappa \frac{k_{B}T}{h}e^{-(\Delta G^{\neq })/RT}\;$$

where *σ* is the reaction symmetry number [29,30], *κ* contains the tunnelling corrections calculated using the Eckart barrier [35], *k_B_* is the Boltzmann constant, *h* is the Planck constant and Δ*G*^≠^ is the Gibbs free energy of activation.

The reaction barriers of single electron transfer (SET) reactions in media were determined using the Marcus theory [40,41]. The equations used to calculate the Gibbs free energy change of reaction Δ*G*^≠^ for the SET pathway are

2.2 $$\Delta G_{\mathrm{SET}}^{\neq }=\;\frac{\lambda }{4}{\left(1+\frac{\Delta G_{\mathrm{SET}}^{0}}{\lambda }\right)}^{2}\;$$

and

2.3 $$\lambda \approx \Delta E_{\mathrm{SET}}\;-\Delta G_{\mathrm{SET}}^{0}\;$$

where Δ*E*_SET_ is the non-adiabatic energy difference among reactants and vertical products for SET, and $\Delta G_{\mathrm{SET}}^{0}$ is the standard Gibbs free energy change of the reaction [42,43].

A correction was applied to rate constants that were close to the diffusion limit [11]. The apparent rate constants (*k*_app_) for an irreversible bimolecular diffusion-controlled reaction were computed using the Collins–Kimball theory in solvents at 298.15 K [44]; the steady-state Smoluchowski rate constant (*k*_D_) was estimated using the literature [11,45],

2.4 $$k_{\mathrm{app}}=\frac{k_{\mathrm{TST}}k_{D}}{k_{\mathrm{TST}}+k_{D}\;}$$

and

2.5 $$k_{D}=4\pi R_{AB}D_{AB}N_{A}$$

*D_AB_* = *D_A_* + *D_B_* (*D_AB_* is the mutual diffusion coefficient of the reactants *A* and *B*) [44,46], where *D_A_* or *D_B_* is determined using the Stokes–Einstein formulation (2.6) [47,48].

2.6 $$D_{A\;or\;B}=\;\frac{k_{B}T}{6\pi_{\eta }a_{A\;or\;B}}\;.$$

*η* is the viscosity of the solvents (i.e. *η*(pentyl ethanoate) = 8.62 × 10 ^−4^ Pa s, *η*(H_2_O) = 8.91 × 10 ^−4^ Pa s) and *a* is the radius of the solute.

To avoid over-penalizing entropy losses in solution, the solvent cage effects were added using Okuno's adjustments [49], which were modified with the free volume theory according to the Benson correction [11,50–52].

For species with numerous conformers, all of them were energy minimized, with the lowest electronic energy conformer being included in the study. The existence of only one single imaginary frequency was a defining feature of all transition stages. To verify that each TS is accurately related to the pre-complex and post-complex, intrinsic coordinate calculations were completed. The calculations were carried out using Gaussian 09 software [53].

## 3. Results and discussions

### 3.1. The gas phase evaluation

Following the established protocol [19,54], the antioxidant activity of **7OGT** was first evaluated according to the three main radical scavenging mechanisms: sequential electron transfer followed by proton transfer (SETPT), formal hydrogen transfer (FHT) and sequential proton loss followed by electron transfer (SPLET). In the two-step reactions such as the SETPT and SPLET pathways, the first step reaction (i.e. SET and proton loss (PL) for the SETPT and SPLET pathways, respectively) normally has the higher activation energy, with the exception of the proton dissociation of acidic moieties in water that is considered separately. Therefore, the thermochemical parameters i.e. proton affinity (PA), bond dissociation energy (BDE) and ionization energies (IE) that characterize the PL, FHT and SET reactions, respectively, were computed for all relevant bonds of **7OGT** with the M06-2X/6-311++G(d,p)//M06-2X/6–31+G(d) method in the gas phase [21]. The results are presented in table 1. The results showed that the BDE values of the C−H range from 84.3 to 99.0 kcal mol^−1^, while the BDEs are 73.9–85.3 kcal mol^−1^ for O-H bonds. This suggests that the hydroxyl groups are the thermodynamically preferred sites of activity via the hydrogen transfer reaction. The lowest BDE values were presented at the O4' − H bond (73.9 kcal mol^−1^) and the O13−H bond (77.1 kcal mol^−1^). These sites are believed to play a key role in **7OGT**'s radical scavenging activity via the FHT mechanism. According to this data, **7OGT** has lower BDE(O−H) values than e.g. vanillic acid (85.2 kcal mol^−1^), [16] puerarin (87.3 kcal mol^−1^), [54] resveratrol (83.9 kcal mol^−1^), [55] or viniferifuran (82.7 kcal mol^−1^) [56]. This suggests that **7OGT** could exhibit faster antiradical activity (following the FHT mechanism) than these natural antioxidants.

**Table 1.** The calculated thermodynamic parameters (BDEs, PAs and IEs, in kcal mol^−1^) and the ΔG^o^ of HOO^**•**^ + **7OGT** reaction.

positions	FHT		PL		SET	
	BDE	ΔG^o^	PA	ΔG^o^	IE	ΔG°
C2 − H	84.3	−2.3			178.8	156.4
C3 − H	99.0	12.5				
C4 − H	86.2	0.3				
O5 − H	85.3	−0.3	345.7	193.1		
O12 − H	83.4	−2.2	353.6	200.6		
O13 − H	77.1	−8.4	340.0	187.4		
O3' − H	80.5	−5.0	341.7	189.1		
O4' − H	73.9	−11.3	341.4	188.7		

As shown in table 1, the lowest IE and PA values are significantly higher (about 2.42 and 4.60 times, respectively) than those of the BDE. Therefore, the antiradical activity of **7OGT** is expected to favour the FHT mechanism in lipid media. The calculated Gibbs free energy changes (Δ*G*°) of the **7OGT** + HOO^•^ reaction via the main mechanisms: FHT, PL, which is the first step of SPLET, and SET as the first step of the SETPT suggest that the FHT reaction is spontaneous (Δ*G*° < 0) for most of the sites, apart from the C3(4)H−bonds; however, the PL and SET reactions are not spontaneous (Δ*G*° > 0) in any cases. The Marcus theory was also used to estimate the reaction barriers of the SET reaction in the gas phase [40,41]; however, this reaction was negative in the studied conditions (Δ*G*^≠^_(SET)_ = 155.6 and *λ* = 21.2 kcal mol^−1^). Thus kinetics of HOO^•^ + **7OGT** reaction were computed following the FHT mechanism at the positions that yielded negative Δ*G*°.

**Figure 2.**
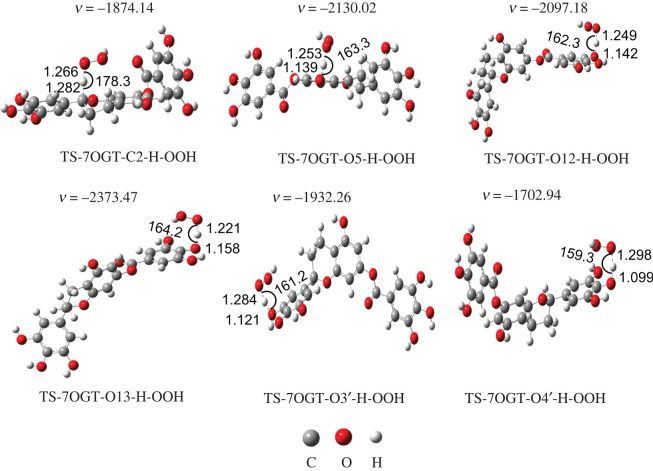
The FHT TSs between the **7OGT** and HOO^**•**^ radical.

Kinetic studies of the HOO^•^ + **7OGT** reaction were performed following the QM-ORSA protocol with the M06-2X/6-311 ++ G(d,p) method [11,27,28], and results are presented in table 2 and figure 2.

**Table 2.** Calculated ΔH, ΔG^≠^ (kcal mol^−1^), tunnelling corrections (*κ*) and *k*_Eck_ (M^−1^ s ^−1^), branching ratios (*Γ*, %) at 298.15 K for the HOO^**•**^ + **7OGT** reaction.

reactions	ΔH	ΔG^≠^	*κ*	k_Eck_	*Γ*
**7OGT** − C2 − H + HOO^**•**^	7.3	16.6	36.4	1.51 × 10^2^	0.0
**7OGT** − O5 − H + HOO^**•**^	3.7	13.5	23.1	1.93 × 10^4^	0.0
**7OGT** − O12 − H + HOO^**•**^	3.9	13.4	46.8	4.23 × 10^4^	0.0
**7OGT** − O13 − H + HOO^**•**^	4.3	14.6	53.3	6.63 × 10^3^	0.0
**7OGT** − O3′ − H + HOO^**•**^	2.6	12.3	19.3	1.13 × 10^5^	0.0
**7OGT** − O4′ − H + HOO^**•**^	−2.0	7.4	19.6	4.58 × 10^8^	100.0
*k*_overall_	4.58 × 10^8^

The energy barriers for the **7OGT** + HOO^•^ reaction following the FHT pathway are within the range of −2.0 to 7.3 kcal mol^−1^ (table 2). The O4' − H + HOO^•^ reaction had the lowest barrier height with Δ*H* = −2.0 kcal mol^−1^. It can be affirmed that the HOO^•^ scavenging activity of the O4′ − H bond is the highest among all of the studied bonds. The HOO^•^ trapping activity of **7OGT** is mainly due to the H-abstraction of the O4′−H bond (Δ*G*^≠^ = 7.4 kcal mol^−1^; *k*_Eck_ = 4.58 × 10^8^ M^−1^ s^−1^; *Γ* = 100%). The activation Gibbs energies (Δ*G*^≠^) range 7.4−16.6 kcal mol^−1^, while the *κ* values vary 19.3−53.3. Thus, the *κ* values play an important role in the rate constants of the hydroperoxyl antiradical activity of the **7OGT**. This result is consistent with previous studies on phenolic compounds [22,27]. The calculated results suggest that the HOO^•^ trapping activity of **7OGT** is defined by the FHT reaction at the O4′ position; therefore, this reaction will be further analysed in physiological media.

### 3.2. The radical scavenging activity of 7-O-Galloyltricetifavan in physiological environments

**Figure 3.**
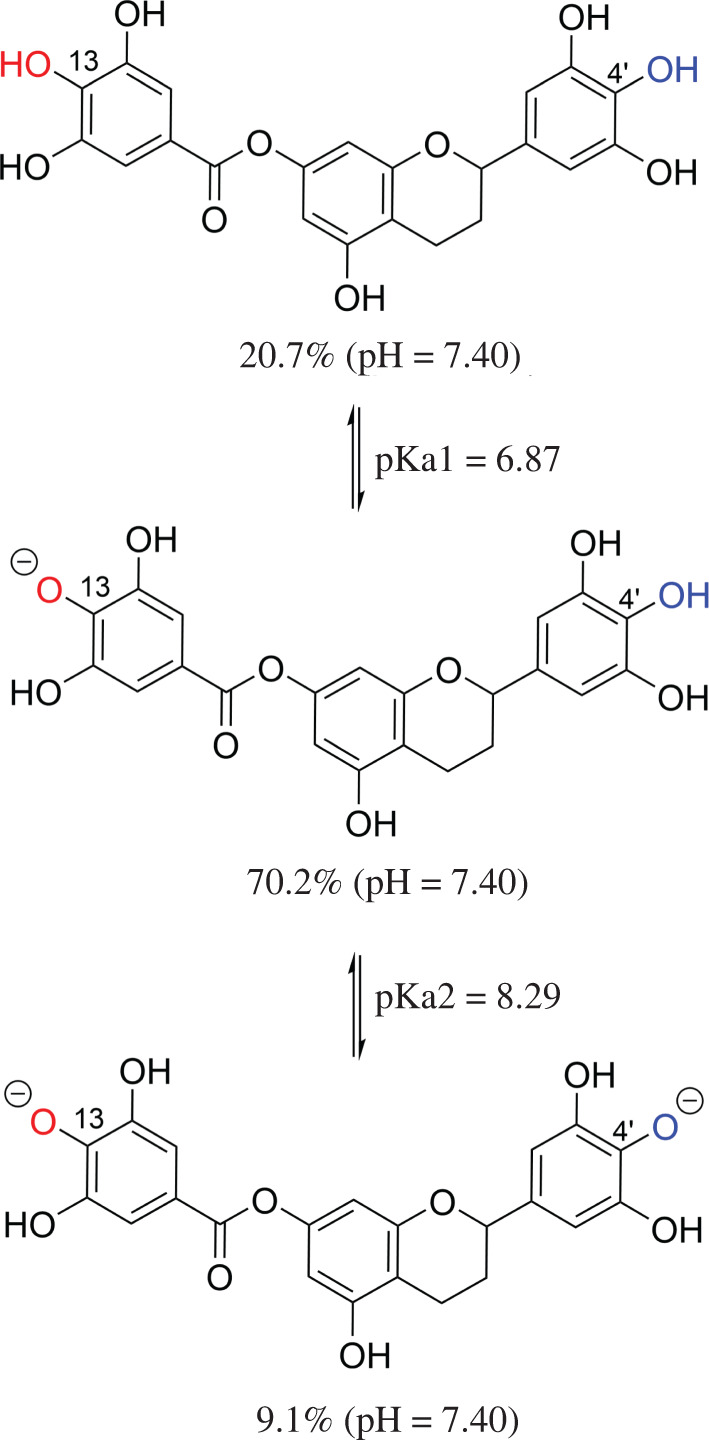
The deprotonation of **7OGT.**

Previous research has shown that the antiradical activity of phenolic compounds in aqueous solutions is dominated by anion states [37,38]. The protonation states of **7OGT** were investigated at physiological pH to discover potential radical scavenging mechanisms [16,38,57]. Based on the calculated data [58], p*K*_a1_ and pK_a2_ values were 6.87 and 8.29, respectively (figure 3). Therefore, in water at pH = 7.4, three states, including neutral (H_2_A: 20.7%), anion (HA^−^: 70.2%) and dianion (A^2−^: 9.1%) will be used for studying the radical scavenging activity, whereas the neutral state will be considered in the lipid medium (pentyl ethanoate solvent).

The calculated results in the vacuum suggest that the HOO^•^ antiradical activity in non-polar media follows the hydrogen transfer mechanism at the O4′ − H bond. Thermodynamic evaluation in pentyl ethanoate and water (electronic supplementary material, table S2) did not differ at the most likely site of activity (BDE = 74.6 and 78.9 kcal mol^−1^ and Δ*G*° = −10.7 and −9.6 kcal mol^−1^ in the lipid and water media, respectively) for the neutral state (H_2_A), however, the PL and SET pathways of this state are not spontaneous in either media (Δ*G*° > 0). Previous studies showed that for a compound containing acidic moieties the SET reaction of the dissociated states should be also considered in the aqueous solution [16,18,21,37,54,57]. Thus, the kinetics of the radical scavenging activity of **7OGT** against HOO^•^ radical in physiological media were carried out following equations (3.1) and (3.2) below, and the results are presented in table 3.

**Table 3.** ΔG^≠^ (kcal mol^−1^), *λ*, *κ, k*_app_, *k*_f_, *k*_overall_ (M^−1^ s^−1^) and *Γ* (%) of the **7OGT** + HOO^**•**^ reaction in the physiological media.

mechanisms		pentyl ethanoate				water					
		ΔG^≠^	*κ*	*k*_app_	*Γ*	ΔG^≠^	*κ*	*k*_app_	*f*	*k*_f_^b^	*Γ*
SET	H_2_A	113.2	19.7	7.10 × 10^−71^	0.0	31.1	19.0^a^	9.60 × 10^−11^	0.207	1.99 × 10^−11^	0.0
	HA^−^					9.0	17.1^a^	1.40 × 10^6^	0.702	9.83 × 10^5^	0.4
	A^2−^					4.3	18.2^a^	2.90 × 10^9^	0.091	2.64 × 10^8^	99.6
FHT	H_2_A	14.2	59.9	1.40 × 10^4^	100.0	15.8	587.0	9.60 × 10^3^	0.207	1.99 × 10^3^	0.0
*k*_overall_				1.40 × 10^4^						2.65 × 10^8^	

^a^*λ*.

^b^*k*_f_ = *f*.*k*_app_; *k*_overall_ = ∑*k*_f_(_kapp_).

Lipid medium,

3.1 $$k_{\mathrm{overall}}=k_{\mathrm{app}}(\mathrm{FHT}(O4^{\prime }-H)-\mathrm{neutral}).$$

Water at physiological pH,

3.2 $$k_{\mathrm{overall}}=k_{f}\;(\mathrm{FHT}\;(O4^{\prime }-H)-\mathrm{neutral})+k_{f}\;(\mathrm{SET}-\mathrm{anion})+k_{f}\;(\mathrm{SET}-\mathrm{dianion}).$$

According to the results (table 3), the HOO^•^ + **7OGT** reaction in the aqueous solution (*k*_overall_ = 2.65 × 10^8^ M^−1^ s^−1^) is approximately 10^4^ times faster than that (*k*_overall_ = 1.40 × 10^4^ M^−1^ s^−1^) in the lipid medium. The SET of dianion A^2−^ plays a principal role (*k*_f_ = 2.64 × 10^8^ M^−1^ s^−1^, *Γ* *=* 99.6%) in the HOO^•^ antiradical activity of **7OGT** in the aqueous solution. Compared with typical antioxidants indicated that the HOO^•^ scavenging activity of **7OGT** is faster than those of Trolox (approx. 2000 times, *k* = 1.30 × 10^5^ M^−1^ s^−1^), [24] *trans*-resveratrol (approx. five times, *k* = 5.62 × 10^7^ M^−1^ s^−1^) [55], ascorbic acid (approx. two times, *k* = 1.00 × 10^8^ M^−1^ s^−1^) [11], ramalin (approx. 1692 times, *k* = 1.56 × 10^5^ M^−1^ s^−1^) [57], deoxynimbidiol (approx. 1.5 times, *k* = 1.69 × 10^8^ M^−1^ s^−1^) [24] and 8-hydroxyconiothyrinone B (approx. 4.5 times, *k* = 5.80 × 10^7^ M^−1^ s^−1^) [18]. Hence, **7OGT** is one of the most excellent natural antioxidants in polar environments.

## 4. Conclusion

The hydroperoxyl antiradical activity of **7OGT** was successfully evaluated by computational chemistry. The results showed that the antiradical activity of the **7OGT** in non-polar media such as gas phase and pentyl ethanoate follows the FHT reaction (*k* = 4.58 × 10^8^ and 1.40 × 10^4^ M^−1^ s^−1^, respectively). **7OGT** also presented excellent antiradical activity (*k*_overall_ = 2.65 × 10^8^ M^−1^ s^−1^) in the aqueous solution. The HOO^•^ radical scavenging of 7OGT is faster than that of typical antioxidants such as *trans*-resveratrol, Trolox, deoxynimbidiol*,* ramalin, 8-hydroxyconiothyrinone B and ascorbic acid. Thus, **7OGT** is one of the most potent natural antioxidants identified thus far in polar environments.

## Data Availability

Data are available at the Dryad Digital Repository: https://doi.org/10.5061/dryad.jh9w0vtcq [59].
